# Physiological risk factors for cardiovascular disease in middle-aged (40-60 year) adults and their association with dietary intake, Northern Iran

**DOI:** 10.22088/cjim.10.1.55

**Published:** 2019

**Authors:** Simin Mouodi, Seyed Reza Hosseini, Robert Graham Cumming, Ali Bijani, Haleh Esmaeili, Reza Ghadimi

**Affiliations:** 1 Social Determinants of Health Research Center, Health Research Institute, Babol University of Medical Sciences, Babol, Iran; 2 School of Public Health, University of Sydney, Sydney, Australia; 3 Health Research Institute, Babol University of Medical Sciences, Babol, Iran

**Keywords:** Diet, Cardiovascular diseases, Overweight, Hyperlipidemia

## Abstract

**Background:**

Considering the importance of healthy diet in the protection of cardiovascular diseases (CVD), this research aimed to assess the physiological risk factors for CVD in middle-aged adults and their association with dietary intake in the north of Iran.

**Methods:**

In this cross-sectional study conducted in the years 2016-2017 among the adults aged 40-60 years living in Amirkola, Babol, the participants´ physical activity, dietary intake, body mass index, fat mass, blood pressure, fasting blood glucose and serum lipid profile were reported. The International Physical Activity Questionnaire was used to assess physical activity and a structured 24-hour food-recall questionnaire was used to assess the participants´ dietary intake. Consumed foods and beverages on two separate days were analyzed and transcribed in 10 food groups (gram).

**Results:**

Two hundred and ninety-one persons (96.7%) had complete participation, 96.2% with at least one CVD risk factor and 75.9% had overweight or obesity; 33% with hypertension; 6.2% had high fasting blood glucose; 64.6% with hypercholesterolemia, 47.8% had hypertriglyceridemia, and 8.9% with low HDL. Mean daily intake values of carbohydrate, protein and fat were all higher than dietary reference intakes for adults. Physical activity less than 1500 MET-minutes per week was significantly associated with having three or more physiological risk factors for CVD [adjusted odds ratio: 2.04 (1.08-3.85)] (P=0.029).

**Conclusion:**

Most of the middle-aged adults in this region had at least one of the physiological risk factors for CVD and daily intakes of carbohydrate and protein were higher than dietary reference intakes (DRIs) for adults.

Cardiovascular diseases (CVD) are the most important contributors to the global NCD burden and the leading cause of deaths around the world (1). World Health Organization reported that an estimated 17.7 million people died from CVDs in 2015, representing 31% of all global deaths. Of these deaths, an estimated 7.4 million were due to coronary heart disease and 6.7 million were due to stroke .Over three quarters of CVD deaths take place in low- and middle-income countries (2). In previous studies, the risk factors for CVD have been divided into three categories: non-modifiable risk factors (such as age, gender, family history of premature CVD and race); behavioral risk factors (such as smoking, unhealthy diet, and inadequate physical activity); and physiological risk factors (especially hypertension, diabetes, overweight and hyperlipidemia) (3, 4).

A healthy diet helps protect against non-communicable diseases (NCDs), including heart diseases, stroke, cancer and diabetes mellitus (5-10). Unhealthy diet and physical inactivity are leading global risks to health (11). A report from a workshop convened by the World Heart Federation demonstrated five important recent changes in food consumption behaviors across the world, even in low- and middle- income countries: 1- a shift to refined carbohydrates – refined grains and added sugars; 2- the increasing intake of vegetable oils, including processed vegetable oils, and a decline in consumption of animal fats; 3- the increasing global consumption of meat; 4- the marked growth of purchases of all packaged foods and beverages; 5- inadequate intake of fruit and vegetables (12). Sugar, salt and especially fat consumption from processed foods have been rapidly increasing in the lower-middle and upper-middle-income countries of Asia and these dietary changes are the most important factors for increasing overweight and obesity in Asian countries (13) such as Iran. Multiple studies have been conducted in a variety of countries in recent years to explore the impact of food consumption on CVD (14-16). Considering the importance of sociocultural factors for food consumption patterns in different regions (17, 18) and the correlation of dietary pattern in each region with the extent of CVD (19-22), this study was conducted to evaluate the current situation of physiological risk factors for cardiovascular disease in apparently healthy middle-aged (40-60 years) adults and their association with dietary intake in the North of Iran. 

## Methods

This analytic study, carried out as a cross-sectional research, is a part of a population-based research which has been approved by the Ethics Committee of Babol University of Medical Sciences with approval code Mubabol.Rec.1394.45. The study was conducted during 2016-2017 among the adults aged 40-60 years living in Amirkola, Babol, North of Iran. Detailed description of the method related to this study has been reported in previous manuscript (23). Exclusion criteria were: pregnant or breastfeeding women; individuals with physical or mental disability; and self- reported medical history of diabetes or hyperlipidemia causing the patient to have a specific diet or to take hypoglycemic and/or lipid lowering agents. Reported data in this study include demographic characteristics (age, gender, education level, marital status and living region), participants´ physical activity, dietary intake, body mass index, fat mass, blood pressure, fasting blood glucose and serum lipid profile. Details of the sampling procedure and criteria for study population recruitment have been described elsewhere (23). In brief, we used various methods to invite and recruit middle-aged population in the study region. Simple random sampling was used to select the households in which a person in 40-60 years age- group was living. Sampling continued until a population of almost equal size of men and women in the age groups of 40-49 and 50-60 years old have been entered in the study.

The International Physical Activity Questionnaire (IPAQ) was used to assess physical activity (PA). Validity and reliability of the Iranian version have been confirmed (24). This questionnaire includes four parts (1. activities at work; 2. housework, gardening and caring for family; 3. PA in transportation; and 4. PA in recreation, sport and leisure time) and asks about the intensity and time a person spent being physically active in the last 7 days. For each domain, participants recorded the number of days and time spent each day undertaking vigorous and moderate-intensity activities separately, along with the time spent walking. Vigorous physical activities are defined as activities that take hard physical effort and make a person breathe harder than normal such as lifting heavy things, digging, heavy construction work, or climbing stairs. Moderate activities are defined as activities that take moderate physical effort and make them breathe somewhat harder than normal such as carrying light loads. These values (vigorous activity, moderate activity, and walking) were used to calculate the PA levels, as specified in the official IPAQ instruction manual. Each type of activity was weighted by its energy requirements as MET (metabolic equivalent of task). Total MET-minutes per week was calculated by multiplying the MET value of a particular activity by 3.3 for walking, 4.0 for moderate PA and 8.0 METs for vigorous PA. Vigorous and moderate activities were defined as those lasting for at least 10 minutes continuously (24). Questions related to time spent sitting were excluded from analysis. 

To assess dietary intake, all participants were interviewed on two separate days (one usual day and one weekend day) using structured 24-hour food-recall questionnaires. The mean of these two days of food records was used to determine the participant´s dietary intake. This questionnaire is a valid tool to assess dietary intake in the study population and has been used in different studies in Iran and other countries (25-28). The participants recorded a detailed diary of foods and beverages consumed in the previous day from early morning until midnight. They were instructed to record their food diary according to standard serving size (26). In this way, two experienced dieticians interviewed and gathered information about food recipes, preparation method, ingredients and quantity of food intake. Dietary records were analyzed using Nutritionist IV software and transcribed in 10 food groups (gram) included: 1.whole grains; 2.dairy products; 3. vegetables; 4. fruits; 5. legumes, nuts and seeds; 6. solid fat and liquid oil; 7. meats, poultry, fish and egg; 8. sugar and sugar-sweetened products (soft drinks, any kind of jam and so on); 9.snack and dessert; 10.tea and coffee. Furthermore, total daily energy (kilocalorie), carbohydrate, fat and protein intake (gram), and the proportion of daily energy from carbohydrate, fat and protein were calculated. Dietary values were compared with Dietary Reference Intakes (DRIs), acceptable macronutrient distribution ranges developed by The National Academies, The Institute of Medicine, Food and Nutrition Board, USA, Last updated 7/27/2017, presented in Appendix 1 (29). In order to determine the influence of daily intake of different food groups on presence of three or more CVD risk factors, the third quartile of the data (gram/day) was considered as a cutoff point. Weight and height were measured and body mass index (BMI) was calculated as weight (in kilogram) divided by height^2^ (in meters) and was classified according to World Health Organization (WHO) recommended cutoff- points: 18.5-24.9 kg/m2 as normal, 25-29.9 kg/m2 overweight and ≥30kg/m2 obese. BMI values in the range of 30-34.9 was classified as obesity class I; 35-39.9 as class II and ≥40 as obesity class III (30). 

Blood pressure was measured with the participant in a sitting position, using a digital sphygmomanometer (Omron M-6 brand). Fat mass percentage was measured using a hand-to-foot bioelectrical impedance analysis technique with a digital body fat calculator (Omron Company: BF511 Model). This technique has been reported as a simple, quick and non-invasive method which can give reliable measurements of body composition with minimal intra- and inter-observer variability. The results are available immediately and reproducible with <1% error on repeated measurements (31). Early morning venous blood samples (5 mL) were collected after fasting for at least 12 hours to assess fasting blood sugar (FBS), total cholesterol, high density lipoprotein (HDL) cholesterol, low density lipoprotein (LDL) cholesterol, total cholesterol and triglycerides (TG) levels. These values were measured using Pars Azmun kits via autoanalyzer respons®910 DiaSys system. All laboratory tests were conducted in a single laboratory which undertook external quality control. 

According to the National Cholesterol Education Program Adult Treatment Panel III Report, cutoff points for fasting blood glucose are considered to be less than 126; total serum cholesterol <200; triglyceride<150; and LDL level less than 100 mg/dL. Serum TG levels in the range of 150-199 was classified as borderline-high TG, 200-499 as high TG and >500 mg/dL as very high TG categories. HDL cholesterol <40 mg/dL was considered as low HDL level (32). High systolic or diastolic blood pressure (≥140/90 mm/Hg), fasting blood glucose ≥126 mg/dL, body mass index ≥25 kg/m2 and abnormal serum lipid profile (total serum cholesterol ≥200, triglyceride ≥150, HDL<40 and LDL ≥ 100 mg/dL) has been considered as physiological risk factors for cardiovascular disease (3, 4). 

Sample size was determined by considering confidence level of 95%, p=0.5 (prevalence of physiological risk factors for cardiovascular disease) and d=0.06 for a sample comprised of 267 individuals (33); in addition, taking a possible loss to follow-up into account, a safety margin of 10-15% was used (34). Data analysis was performed with SPSS software Version 17. The Kolmogorov-Smirnov test was used to evaluate the normal distribution of quantitative data. Chi-square was used to compare categorical variables (education level, marital status, living region, the presence of overweight, obesity, high blood pressure, high fasting blood glucose, abnormal serum lipid profile and the number of physiological risk factors for CVD) between two sexes. T-test was used to compare quantitative data (age, blood pressure, total physical activity, the amount of daily intake of each food group, daily energy and nutrients intake) between men and women and Pearson´s correlation coefficient was calculated to assess the correlation between the number of physiological risk factors for CVD and daily intake of the ten food groups. The logistic regression model was performed to determine the association of physical activity and dietary intake of different food groups with presence of three or more CVD risk factors. Crude and adjusted odds ratio (OR) of age, gender, daily intake of food groups and physical activity and their 95% confidence intervals were calculated. The p-value less than 0.05 was considered as the significant level. 

## Results

Three hundred and one individuals were enrolled in the study. Of these, 291 (96.7%) persons had complete participation, filling out two separate 24-hour food-recall questionnaires. One-hundred and fifty one (51.9%) were females and 140 (48.1%) were males. Most of them were married; had education level less than high school diploma; and were living in urban regions. Demographic characteristics, anthropometric, physical activity, serum glucose, lipid profile and dietary intake of these 291 participants divided into two sexes are presented in table1. This table shows that mean BMI, fat mass, total physical activity, food and nutrient intakes, serum TG and HDL values were statistically significantly different between men and women (p<0.05). Mean daily intake values of carbohydrate, protein and fat were all higher than dietary reference intakes for adults.

**Table1:** Demographic and anthropometric characteristics, blood pressure, physical activity, dietary intake, fasting blood sugar and serum lipid profile of 40-60 years adults, Amirkola, northern Iran

**Variables**	**Female (n=151)** **Mean±SD**	**Male (n=140)** **Mean±SD**	**Total (n=291)** **Mean±SD**	**P-value**
Age (years)	49.2±5.1	50.4±5.6	49.7±5.4	0.057
**Level of education** Less than diploma Diploma Academic education	90 (59.6) 48 (31.8) 13 (8.6)	81 (57.9) 35 (25.0) 24 (17.1)	171(58.8) 8 (28.5) 37 (12.7)	0.068
**Marital status** Married Not-married (single or widow or divorced)	139 (92.1) 12 (7.9)	139 (99.3) 1 (0.7)	278 (95.5) 13 (4.5)	0.003
Living region: Urban Rural	148 (98.0) 3 (2.0)	134 (95.7) 6 (4.3)	282 (96.9) 9 (3.1)	0.258
Body Mass Index (kg/m2)	29.2±4.8	27.2±3.8	28.3±4.4	<0.001
Fat mass (percentage)	39.4±6.3	23.2±6.8	31.6±10.4	<0.001
Systolic blood pressure (mm/Hg)	124.6±16.3	125.5±16.9	125.0±16.6	0.631
Diastolic blood pressure (mm/Hg)	80.0±9.9	81.2±10.8	80.6±10.3	0.302
Total physical activity (MET-min/week)	2981.5±3336.7	8279.8±6588.3	5605.5±5932.8	<0.001
Ten food groups daily intake (gram/day): 1.whole grains 2.dairy products 3. vegetables 4. fruits 5. legumes, nuts and seeds 6. solid fat and liquid oil 7. meats, poultry, fish and egg 8. sugar and sugar-sweetened products (soft drinks, honey, any kind of jam and so on) 9.snack and dessert 10.tea and coffee	344.7±142.1 135.1±149.2 229.0±151.5 409.7±232.1 55.6±60.0 14.5±14.9 100.8±71.0 34.2±53.4 25.1±28.4 567.3±395.8	522.7±197.6 192.4±189.5 240.5±158.0 513.1±366.5 68.2±86.4 14.8±16.4 143.9±92.4 44.0±72.5 18.2±25.0 657.3±370.4	430.4±192.6 162.6±171.9 234.5±154.5 459.4±308.1 61.7±74.0 14.6±15.7 121.5±84.9 38.9±63.4 21.8±27.0 610.6±385.8	<0.001 0.004 0.529 0.004 0.148 0.879 <0.001 0.185 0.031 0.047
Total daily energy (kilocalorie)	1830.9±490.8	2561.9±747.6	2182.6±725.5	<0.001
Daily intake of carbohydrate (gram)	287.6±84.6	403.3±116.8	343.3±116.6	<0.001
Daily intake of protein (gram)	66.3±22.1	99.3±37.2	82.2±34.5	<0.001
Daily intake of fat (gram)	46.1±22.3	61.2±30.0	53.4±27.3	<0.001
Proportion of energy from carbohydrate (%)	63.2±8.9	63.4±7.6	63.3±8.3	0.835
Proportion of energy from protein (%)	14.5±3.4	15.5±3.4	15.0±3.4	0.014
Proportion of energy from fat (%)	22.2±7.6	21.1±6.2	21.7±7.0	0.150
Fasting blood glucose (mg/dL)	94.2±14.4	101.1±36.4	97.5±27.5	0.302
Serum TG (mg/dL)	158.3±100.6	190.3±134.3	173.7±118.9	0.022
Serum total cholesterol (mg/dL)	212.5±38.8	222.1±54.7	217.1±47.3	0.083
Serum HDL (mg/dL)	60.0±14.1	53.5±14.8	56.9±14.7	<0.001
Serum LDL (mg/dL)	116.5±23.3	120.6±29.5	118.5±26.5	0.189

Distribution of physiological risk factors for cardiovascular disease in the study population is presented in table 2. This table shows that 82.1% of women and 69.3% of men were in the range of overweight or obesity; furthermore, overweight or obesity, high fasting blood glucose, high serum triglyceride and low serum HDL level were significantly different between men and women (p<0.05). Only 3.8% of the study population had no physiological risk factors for CVD. 

The correlation between the number of physiological risk factors for CVD and daily intake of the ten food groups was assessed. The results showed that daily intake of fruits (Pearson correlation=0.157, P=0.007) and vegetables (Pearson correlation=0.115, P=0.051) had significant correlations with the number of physiological risk factors for CVD. Other food groups did not have significant correlations with number of physiological risk factors for CVD (p>0.05). To assess the influence of different examined variables (age, gender, physical activity and daily intake of food groups) and presence of three or more physiological risk factors for CVD by using logistic regression model revealed that daily intake of no food group (gram/day) had significant association with having at least three physiological risk factors for CVD (p>0.05). Only physical activity less than 1500 MET-minutes per week (25) was significantly associated with having at least three of the mentioned physiological risk factors for CVD (p=0.029) (table 3).

**Table 2:** Distribution of physiological risk factors for cardiovascular disease in 40-60 years adults, Amirkola, Northern Iran

**Variable**	**Female (n=151)** **Number (percent)**	**Male (n=140)** **Number (percent)**	**Total (n=291)** **Number (percent)**	**P-value**
**Body Mass Index (kg/m2):**				
18.5-24.9 25-29.9 30-34.9 35-39.9 ≥40	27 (17.9) 68 (45.0) 42 (27.8) 10 (6.6) 4 (2.6)	43 (30.7) 64 (45.7) 28 (20.0) 5 (3.6) 0 (0)	70 (24.1) 132 (45.4) 70 (24.1) 15 (5.2) 4 (1.4)	0.009
**Overweight or obesity:**				
Yes No	124 (82.1) 27 (17.9)	97 (69.3) 43 (30.7)	221 (75.9) 70 (24.1)	0.010
**High blood pressure:**				
Yes No	48 (31.8) 103 (68.2)	48 (34.3) 92 (65.7)	96 (33.0) 195 (67.0)	0.651
**High fasting blood glucose:**				
Yes No	5 (3.3) 146 (96.7)	13 (9.3) 127 (90.7)	18 (6.2) 273 (93.8)	0.035
**Abnormal serum lipid profile:**				
Total serum cholesterol ≥200mg/dL Serum triglyceride ≥150 mg/dL Serum HDL<40 mg/dL Serum LDL ≥ 100 mg/dL Total abnormal lipid profile: Yes No	93 (61.6) 66 (43.7) 8 (5.3) 112 (74.2) 126 (83.4) 25 (16.6)	95 (67.9) 73 (52.1) 18 (12.9) 106 (75.7) 125 (89.3) 15 (10.7)	188 (64.6) 139 (47.8) 26 (8.9) 218 (74.9) 251 (86.3) 40 (13.7)	0.264 0.150 0.024 0.762 0.148
**Total number of physiological risk factors for CVD:**				
One risk factor Two risk factors Three risk factors Four risk factors No risk factor	30 (19.9) 76 (50.3) 39 (25.8) 1 (0.7) 5 (3.3)	32 (22.9) 60 (42.9) 37 (26.4) 5 (3.6) 6 (4.3)	62 (21.3) 136 (46.7) 76 (26.1) 6 (2.1) 11 (3.8)	0.361

**Table 3:** Physiological risk factors for CVD in 40-60 years adults and their association with dietary intake and physical activity

Variable	**Physiological risk factors for CVD**		**Crude odds ratio for 0/1(95% CI)**	**P-value**	**Adjusted odds ratio (95%CI)**	**P-value**
	**< 3 risk factors** **(N=209)** **N (%)**	**≥ 3 risk factors** **(N=82) ** **N (%)**				
**Gender**						
Female (0) Male (1)	111 (53.1) 98 (46.9)	40 (48.8) 42 (51.2)	1.2 (0.71-1.98)	0.506	1.57 (0.86-2.89)	0.146
**Age (years)**						
< 50 (0) ≥ 50 (1)	105 (50.2) 104 (49.8)	33 (40.2) 49 (59.8)	1.5 (0.89-2.52)	0.124	1.50 (0.88-2.56)	0.133
**Daily vegetables intake (325 gram/day)**						
No Yes	158 (75.6) 51 (24.4)	60 (73.2) 22 (26.8)	1.14 (0.64-2.03)	0.667	1.16 (0.64-2.12)	0.624
**Daily total fat intake (20 gram/day)**						
No Yes	154 (73.7) 55 (26.3)	65 (79.3) 17 (20.7)	0.73 (0.39-1.36)	0.321	0.83 (0.44-1.59)	0.580
**Daily whole grains (550 gram/day)**						
No Yes	155 (74.2) 54 (25.8)	63 (76.8) 19 (23.2)	0.87 (0.48-1.58)	0.637	0.83 (0.42-1.63)	0.585
**Physical activity (1500 MET-minute/ week)**						
No Yes	165 (78.9) 44 (21.1)	56 (68.3) 26 (31.7)	1.74 (0.98-3.08)	0.506	2.04 (1.08-3.85)	0.029

## Discussion

In our study in northern Iran, 96.2% of supposedly healthy 40-60 years old adults had at least one of the physiological risk factors for CVD and 75.9% had overweight or obesity; 33% with high blood pressure; 6.2% had high fasting blood glucose; 64.6% with hypercholesterolemia, 47.8% had hypertriglyceridemia, and 8.9% with low HDL. Hajian-Tilaki examined a sample of 1000 adults aged 20-70 years in urban areas of Babol, North of Iran and demonstrated that only 7.8% of men and 2.7% of women in this region had no assessed CVD risk factors (abnormal serum lipid profile, abdominal obesity, high blood pressure and fasting blood glucose) (35). A greater amount of CVD risk factors in our research can be attributed to recruitment of higher aged adults in our study in comparison with Hajian´s study. Jafari-Adli reported in his systematic review that the prevalence of overweight or obesity among normal adult or children population samples in Iran was 27.0-38.5% (36). Moreover, our result has similarity with Bahadoran´s study in Iran, conducted among middle-aged (31-50 years) adults, in which 44.3% had overweight and 29.7% were obese; and the prevalence of hypertriglyceridemia, hypercholesterolemia and hypertension were reported as 37.1%, 35.2% and 16.1%, respectively (37). In Nag´s study in West Bengal of India, conducted among rural adults (aged ≥20 years), 52.5% had high blood pressure, 23.1% high fasting blood glucose, 45.6% hypertriglyceridemia, 11.2% hypercholesterolemia, and 11.6% had low HDL (38). 

In our study, men had a higher serum TG level than women; women had a higher BMI, fat mass and serum HDL level than men. However, men and women did not have a significant difference in the number of CVD risk factors. In Hajian´s study in the North of Iran, a higher prevalence of cardiometabolic risk factors for CVD has been found in females than males (35). In the research of Truthmann in Germany, the prevalence of overweight in adults aged 40-79 years without prior coronary heart disease or stroke was greater in men than women (76.2% versus 60.5%); Nevertheless, obesity (27.2% versus 25.7%) and also elevated total cholesterol (73.4% versus 68.8%) were greater in women than men. The prevalence of most behavioural cardiovascular risk factors has been reported to be higher among men compared to women (40). This difference in results may be attributed to the study population, different demographic and socioeconomic characteristics and specific lifestyle behaviors of each region (40). 

In our study, only 24.1% of 40-60 years adults had normal BMI; most of the study population (75.9%) were overweight or obese, with higher prevalence in women. As Hajian reported, female adults might be more obese than males (35). In a systematic review study, it has been reported that the prevalence of overweight and obesity is very high in most countries of the Eastern Mediterranean region, ranging from 25% to 82%, with a higher prevalence among women (41). The secular trends of overweight and obesity among Iranian adults (25-64 years old) within an 8-year period (1999-2007) showed that the overall prevalence of obesity increased from 13.6% in 1999 to 22.3% in 2007. During these years, the mean body mass index increased from 25.0 to 26.5. The increase in the prevalence of obesity has been observed in both males and females and both urban and rural populations (42). 

In this research, daily intakes of carbohydrate and protein were higher than dietary reference intakes (DRIs) for adults and none of the ten food groups had significant correlations with the number of physiological risk factors for CVD. Mirmiran, in Tehran evaluated the dietary pattern of 2284 people without CVD in the central region of Iran and identified two major dietary patterns, Western and traditional. She reported that a traditional dietary pattern was not associated with incidence of CVD or CVD risk factors; nonetheless, Western dietary pattern, characterized by higher loads of processed meats, salty snacks, sweets, and soft drinks, represented a dietary risk factor for CVD in the Iranian population (43). Esmaillzadeh reported in his research that food intake patterns may explain the high prevalence of CVD risk factors in Iranian women. He demonstrated that higher fiber intake with lower energy and cholesterol intakes was correlated with lower serum triglyceride, total and LDL cholesterol, and fasting plasma glucose concentrations, lower systolic and diastolic blood pressures, and a higher serum HDL-cholesterol concentration (44). Darani Zad in Iran evaluated dietary patterns and associations with biochemical blood profiles and body weight among 400 adults aged 40–60 years and reported that a mixed dietary pattern (including nuts, fruit, olive oil and tea) was associated with healthier lipid profiles (43). Bechthold´systematic review represented food-based priorities to reduce CVD risk. He listed them as to increase consumption of whole grains, fruits, vegetables, legumes, nuts and seeds, and fish; to keep a moderate dairy and vegetable oil intake, and to avoid or reduce consumption of refined grains, sugar-sweetened beverages, and red and processed (sodium-preserved) meats (45). In the northern region of Iran, near the Caspian Sea, a considerable percentage of the population has crop farms and gardens, and daily intake of grains (especially white rice) combined with fatty stewed foods (46, 47) is a common dietary pattern which can have an impact on body weight and CVD risk factors (13) . 

The proportion of energy from fat intake in our study was 21.7%, which is much lower than the World Health Organization’s recommendation that total fat should not exceed 30% of total energy intake, with a shift in fat consumption away from saturated fats to unsaturated fats and towards the elimination of industrial trans fats (48). In our study, only physical activity less than 1500 MET-minutes per week was significantly associated with having three or more physiological risk factors for CVD. Other variables (age, gender and dietary intake of different food groups) did not have a significant impact. Koolhaas suggested that the beneficial impact of physical activity on CVD might outweigh the negative impact of body mass index among middle-aged and elderly people (49). Furthermore, the influence of physical activity on different aspects of CVD such as blood pressure, serum lipid profile, blood coagulation and fibrinolysis and vascular remodeling (50) should be considered, as well as the effect of increasing physical activity on other health behaviors, such as diet and smoking (51). 

The most important strong points of this research include the design of study as a population-based assessment of apparently healthy middle-aged adults and the use of two 24-hour food recall questionnaires to assess the participants´ dietary intake. Seasonal variations in dietary patterns in Iran (52), and changes in dietary and nutrient intake, including fasting, according to important Muslim ceremonies (53) are limitations of this research. Sample size was calculated based on the prevalence of CVD risk factors, and this design reduced the study power. Furthermore, we did not take into account some of the variables such as alcohol, tobacco, and substance abuse which can have correlations with assessed CVD risk factors. 

In conclusion most middle-aged (40-60 years) adults in the northern Iran, in our study region, had one to four physiological risk factors for cardiovascular disease and daily intakes of carbohydrate and protein were higher than dietary reference intakes for adults. We also found that the association of low physical activity with physiological risk factors for CVD was more significant than intake of different food groups. 

**Ethics approval and consent to participate**

All participants provided a written informed consent form. They have been assured that their information would be kept confidential. This research was approved by the Ethics Committee of Babol University of Medical Sciences with registration code Mubabol.Rec.1394.45. 
